# Safety of combination therapy of azilsartan medoxomil and amlodipine: a population-based cohort study

**DOI:** 10.4178/epih.e2025029

**Published:** 2025-05-28

**Authors:** Hyesung Lee, Bin Hong, Chris Tzu-Ting Su, Sungho Bea, Han Eol Jeong, Kyungyeon Jung, Michael Chun-Yuan Cheng, Zoe Chi-Jui Chang, Edward Chia-Cheng Lai, Jongyoung Lee

**Affiliations:** 1 Department of Medical Informatics, Kangwon National University College of Medicine, Chuncheon, Korea; 2 School of Pharmacy, Sungkyunkwan University, Suwon, Korea; 3 School of Pharmacy, Institute of Clinical Pharmacy and Pharmaceutical Sciences, College of Medicine, National Cheng Kung University, Tainan, Taiwan; 4 Division of Pharmacoepidemiology and Pharmacoeconomics, Department of Medicine, Brigham and Women’s Hospital and Harvard Medical School, Boston, MA, USA; 5 DSC Investment, Seoul, Korea; 6 Division of Cardiology, Kangbuk Samsung Hospital, Sungkyunkwan University School of Medicine, Seoul, Korea

**Keywords:** Antihypertensive agents, Combination drug therapy, Hypertension

## Abstract

**OBJECTIVES:**

This study investigated the safety of azilsartan and amlodipine combination therapy versus other angiotensin receptor blockers (ARBs) and amlodipine in patients with hypertension.

**METHODS:**

We conducted a cohort study utilizing healthcare databases from Korea and Taiwan. Patients aged between 18 years and 75 years who were newly prescribed both an ARB and amlodipine within 6 months of hypertension diagnosis were included. Safety outcomes assessed were hypotension, angioedema, acute pancreatitis, hyperkalemia, hypokalemia, toxic liver disease, hepatic failure, nausea and vomiting, and fall-related injury. Hazard ratios (HRs) with 95% confidence intervals (CIs) for each safety outcome associated with azilsartan medoxomil and amlodipine versus other ARBs combined with amlodipine were calculated within a 1:1 propensity score (PS)-matched cohort. Summary HRs across databases were computed using random-effects meta-analysis.

**RESULTS:**

We identified 2,472 eligible patients (1,521 from Korea, 951 from Taiwan) initiating treatment with azilsartan medoxomil and amlodipine, and 671,468 patients (312,322 from Korea, 355,409 from Taiwan) initiating other ARBs with amlodipine. After PS matching, baseline characteristics were well-balanced between treatment groups. During the 180-day follow-up, most adverse outcomes did not occur even once in either group, thus precluding the calculation of HRs. The risk of acute pancreatitis was not significantly different between the azilsartan medoxomil and amlodipine group and the other ARB and amlodipine groups (summary HR, 0.86; 95% CI, 0.14 to 5.37).

**CONCLUSIONS:**

In this population-based cohort study, azilsartan medoxomil combined with amlodipine was not associated with an increased risk of adverse outcomes compared to other ARBs combined with amlodipine.

## GRAPHICAL ABSTRACT

[Fig f3-epih-47-e2025029]

## Key Message

Limited studies have evaluated the long-term safety of combined azilsartan medoxomil and amlodipine therapy. This cohort study found the azilsartan and amlodipine combination therapy was not associated with increased risk of serious adverse events compared to other ARB-amlodipine combinations. These results support the real-world safety of azilsartan-amlodipine caombination therapy in patients with hypertension.

## INTRODUCTION

Hypertension represents a significant global health burden, affecting an estimated 1.3 billion individuals worldwide [1,2]. It is an important risk factor for mortality, causing more than 8 million deaths annually worldwide due to cardiovascular complications such as coronary heart disease, heart failure, and stroke [1]. Guidelines recommend thiazide diuretics, angiotensin‐converting enzyme (ACE) inhibitors, angiotensin receptor blockers (ARBs), and calcium channel blockers (CCBs) as first-line primary agents for hypertension treatment [3]. Moreover, most patients, particularly those with stage 2 hypertension or comorbidities, are advised to initiate treatment with a combination of 2 medications to effectively manage their blood pressure [4].

ARBs and CCBs have independently demonstrated benefits in reducing blood pressure and lowering cardiovascular morbidity and mortality in hypertensive patients [5,6]. Additionally, recent evidence suggests that combining these medications exerts complementary effects on blood pressure control, as they target distinct signaling pathways crucial for vascular regulation [7,8]. Randomized clinical trials (RCTs) have demonstrated that combination therapy with amlodipine (a CCB) and valsartan (an ARB) substantially reduces blood pressure compared to high-dose monotherapy, with significantly more patients achieving blood pressure targets as early as 2 weeks after initiation [9]. Moreover, this combination regimen has exhibited good tolerability and safety, with only mild to moderate adverse events reported [10,11].

Azilsartan medoxomil is a novel, long-acting ARB approved by the U.S. Food and Drug Administration and the European Medicines Agency in 2011 [12,13]. Azilsartan medoxomil has been reported to exhibit more potent antihypertensive effects at the maximum approved dose compared to other commonly used ARBs, such as olmesartan and valsartan [14-16]. Furthermore, RCTs have demonstrated that combination therapy with azilsartan medoxomil and amlodipine is as effective as telmisartan with amlodipine and achieves greater blood pressure reductions compared to amlodipine monotherapy [17,18]. Animal studies suggest that azilsartan medoxomil binds more strongly to its receptor than other ARBs and may dissociate more slowly, resulting in a prolonged duration of action. However, limited studies have evaluated the long-term safety of combined azilsartan medoxomil and amlodipine therapy [19].

Therefore, this study aimed to evaluate the 6-month safety of azilsartan medoxomil combined with amlodipine compared to other ARBs combined with amlodipine among hypertensive patients, using administrative healthcare claims data from Korea and Taiwan.

## MATERIALS AND METHODS

### Data sources

We conducted a distributed study using a common study protocol approach involving 2 representative healthcare databases: Korea’s Health Insurance Review and Assessment Service (HIRA) database from July 1, 2018, to December 31, 2021, and Taiwan’s National Health Insurance Research Database (NHIRD) from January 1, 2013, to December 31, 2020 [20]. Researchers from each location independently implemented data analysis based on the common protocol, which included detailed descriptions of key design elements and analytic parameters.

The HIRA database covers approximately 98% of the Korean population, compiling all medical claims submitted by healthcare providers for reimbursement. This database provides comprehensive information about healthcare services delivered to beneficiaries, including diagnoses, treatments, procedures, surgical histories, and prescribed medications. Diagnoses were coded using the Korean Standard Classification of Diseases, 7th revision (KCD-7), adapted from the International Classification of Diseases, 10th revision (ICD-10). Prescription details included the generic drug names, prescription dates, durations, and administration routes. Prior validation studies compared diagnoses from claims data with hospital or clinic medical records, demonstrating an overall positive predictive value of 82% for diagnoses in the Korean healthcare database [21].

The NHIRD is an anonymized claims database covering approximately 99% of Taiwan’s population (over 23 million individuals). This database includes beneficiaries’ demographic information, ambulatory care claims, inpatient claims, pharmacy prescription records, and registry data on medical facilities and board-certified specialists.

### Study population

We included patients aged between 18 years and 75 years who first received a hypertension diagnosis (ICD-10: I10-I15; ICD-9: 401-405) as a primary diagnosis (e.g., principal diagnosis) between January 1, 2019, and June 30, 2020, in the HIRA database, and between January 1, 2014, and June 30, 2019, in the NHIRD. Patients prescribed both ARBs and amlodipine within 6 months of their initial hypertension diagnosis were included. The cohort entry date was defined as the date of the first prescription for ARB and/or amlodipine. To identify new users of the study medications, patients prescribed ARBs, ACE inhibitors, or CCBs within the 6 months preceding the cohort entry date were excluded.

Patients were then classified into 2 groups based on ARB types prescribed within a 30-day window after cohort entry (i.e., the treatment group assessment window): (1) azilsartan medoxomil+amlodipine group: patients prescribed both azilsartan medoxomil and amlodipine on the cohort entry date or those who added on amlodipine to azilsartan medoxomil that was prescribed at cohort entry within the treatment group assessment window, or vice versa; (2) other ARB+amlodipine group: patients prescribed both ARB (except azilsartan medoxomil) and amlodipine on the cohort entry date or those who added on amlodipine to ARB that was prescribed at cohort entry within the treatment group assessment window, or vice versa. Other ARBs included candesartan, irbesartan, losartan, valsartan, telmisartan, eprosartan, Olmesartan, and fimasartan.

**Figure 1.**
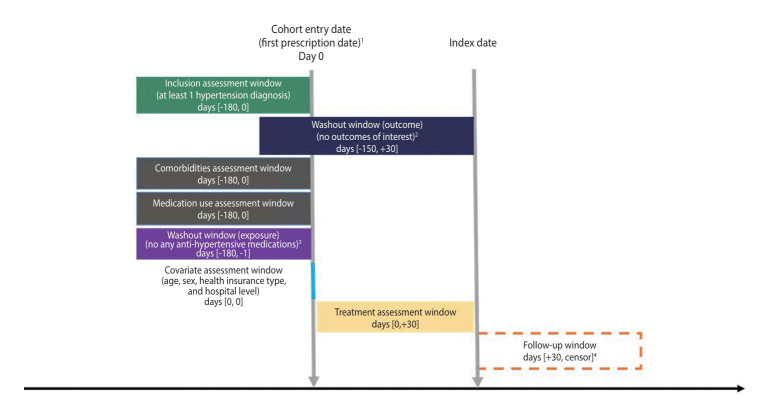
Study diagram. ^1^First prescription date indicates the first prescription date for angiotensin receptor blockers (ARBs), and calcium channel blockers (CCBs). ^2^Outcomes of interest include hypotension, angioedema, acute pancreatitis, hyperkalemia, hypokalemia, toxic liver disease, hepatic failure, nausea and vomiting, and fall-related injury. ^3^Antihypertensive medications include any type of angiotensinconverting enzyme inhibitor, ARB, and CCB. ^4^Censored at earliest occurrence of the study outcome, switching to another group, discontinuation (either ARB or amlodipine), death, or end of the study period.

**Figure 2.**
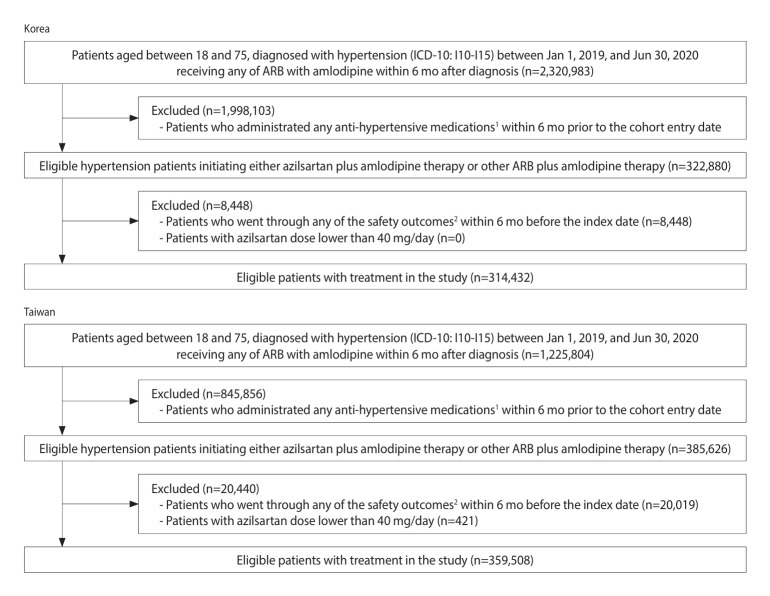
Study flowchart. ACE, angiotensin converting enzyme; ARB, angiotensin receptor blockers; CCB, calcium channel blocker; ICD-10, International Classification of Diseases, 10th revision, Clinical Modification. 1Antihypertensive medications include any type of ACE inhibitor, ARB, and CCB. 2Outcomes included hypotension, angioedema, acute pancreatitis, hyperkalemia, hypokalemia, toxic liver disease, hepatic failure, nausea and vomiting, and fall-related injury.

The index date was defined as the end of the 30-day treatment group assessment window after cohort entry. To ensure incident events, we excluded patients diagnosed with any of the specified safety outcomes within 6 months prior to the index date. Additionally, to maintain consistency across sites, patients prescribed azilsartan medoxomil at 20 mg/day were excluded, since only 40 mg and 80 mg tablets are approved in Korea [22]. Lastly, patients initiating treatment with both azilsartan medoxomil and other ARBs within the assessment window were also excluded. The study design and patient flowchart are illustrated in Figures 1 and 2, respectively.

### Study outcomes and follow-up

We assessed 9 safety outcomes of interest: hypotension, angioedema, acute pancreatitis, hyperkalemia, hypokalemia, toxic liver disease, hepatic failure, nausea and vomiting, and fall-related injury. These outcomes were selected because they represent known or suspected clinically significant adverse events associated with antihypertensive medications [18,23]. To enhance accuracy, outcomes were restricted to primary or secondary diagnoses. Detailed outcome definitions are provided in Supplementary Material 1.

Patients were followed from the index date until the occurrence of a study outcome, switching to another treatment group, discontinuation of either ARB or amlodipine, death, or 180 days after the index date—whichever occurred first—using an as-treated follow-up approach. Continuous exposure was defined as having less than a 30-day gap between the end date of 1 prescription and the start date of the subsequent prescription.

### Potential confounders

Socio-demographic covariates, including age, sex, and health insurance type, were assessed at cohort entry. We also recorded the hospital level where azilsartan medoxomil or another ARB was first prescribed. Hospital levels were categorized into 2 groups: (1) medical centers or regional hospitals, and (2) other institutions such as district hospitals and clinics. The Charlson comorbidity index (CCI) was calculated as an indicator of overall comorbidity burden. Comorbidities diagnosed during the 6 months preceding cohort entry were considered. These comorbidities included acute respiratory illness, chronic liver disease, chronic obstructive pulmonary disease, diabetes, gastrointestinal bleeding, gastroesophageal reflux disease, hyperlipidemia, malignancies, obesity, pneumonia, osteoarthritis, psoriasis, kidney disease, rheumatoid arthritis, urinary tract infections, ulcerative colitis, visual system disorders, atrial fibrillation, coronary artery sclerosis, cerebrovascular disease, peripheral vascular disease, pulmonary embolism, and venous thromboembolism [23]. Additionally, co-medications prescribed within the 6 months before cohort entry were evaluated, including systemic antibacterials, antidepressants, antiepileptics, anti-inflammatory and antirheumatic drugs, antineoplastic agents, antithrombotic drugs, beta-blockers, medications for airway obstruction, medications for acid-related diseases, antidiabetic drugs, immunosuppressants, lipid-lowering drugs, opioids, psychostimulants, and psycholeptics [23]. Specific definitions of comorbidities and co-medications are listed in Supplementary Material 1.

### Statistical analysis

Results are presented as frequencies for categorical variables and as means (standard deviations) or medians (interquartile ranges) for continuous variables. Selected baseline characteristics were compared between the 2 treatment groups, and covariate balance was assessed using the absolute standardized mean difference, where a value greater than 0.1 indicates imbalance.

We estimated the propensity score (PS) for receiving azilsartan medoxomil and amlodipine combination therapy using multivariate logistic regression that included all measured covariates in the model. A 1:1 greedy nearest-neighbor PS matching approach was applied to establish comparability between the 2 groups. Matched pairs were formed using an 8-to-1-digit greedy nearest-neighbor matching algorithm without replacement (i.e., each patient receiving azilsartan medoxomil and amlodipine was matched to the patient receiving another ARB and amlodipine who had the closest PS, with each patient included in matching pairs only once) [24]. Within the PS-matched cohort, we calculated the number of events, person-years, and incidence rate per 1,000 person-years for all outcomes in each treatment group. Cox proportional hazard models were used to estimate hazard ratios (HRs) with 95% confidence intervals (CIs). We then conducted a random-effects meta-analysis to calculate summary HRs, pooling effect estimates across the 2 databases. Statistical analyses were performed using SAS Enterprise Guide version 7.1 (SAS Institute Inc., Cary, NC, USA).

#### Subgroup and sensitivity analyses

We conducted subgroup analyses according to the initial azilsartan medoxomil dose: standard dose (40 mg, as recommended by the Ministry of Food and Drug Safety, Korea) and high-dose (80 mg). The PS was re-estimated, and matching was performed separately within the high-dose and standard-dose groups. Additionally, we evaluated the risk of adverse outcomes specifically among patients who received a combination of high-dose azilsartan medoxomil (80 mg) and high-dose amlodipine (10 mg). Furthermore, we conducted a sensitivity analysis by selecting olmesartan as an alternative active comparator instead of other ARBs, given that azilsartan medoxomil and olmesartan both exhibit stronger antihypertensive effects compared to other ARBs [25]. We also performed sensitivity analysis using PS fine stratification rather than matching, aiming to estimate the average treatment effect. After trimming observations from non-overlapping regions of the PS distribution, patients receiving the study medications were stratified into 50 equal-sized strata based on their PS distributions. In each stratum, patients receiving azilsartan medoxomil and amlodipine were assigned a weight of 1, whereas patients receiving other ARBs and amlodipine were re-weighted according to the proportion of exposed patients within the corresponding stratum. Finally, we conducted an intention-to-treat analysis, in which patients were followed from the index date until the earliest occurrence of a study outcome, death, or the end of a 180-day follow-up period. This approach was intended to minimize concerns regarding informative censoring.

### Ethics statement

This study was approved by the institutional review board of each site (Korea: SKKU 2022-07-018; Taiwan: HREC No. 111-715), and the requirements for informed consent were waived because the data analyses were performed retrospectively using anonymized data. Authors received permission to access the database from the database owners.

## RESULTS

### Study population and baseline characteristics

We identified a total of 673,940 eligible patients: 314,432 from the HIRA database, of whom 1,521 received azilsartan medoxomil and amlodipine and 312,911 received other ARBs combined with amlodipine; and 359,508 from the NHIRD database, of whom 951 received azilsartan medoxomil and amlodipine and 358,557 received other ARBs combined with amlodipine (Figure 2). Before PS matching, although many baseline characteristics were well balanced, patients initiating azilsartan medoxomil and amlodipine were more likely to have prescriptions issued by medical centers or regional hospitals at both study sites (azilsartan medoxomil group: 51.1% vs. other ARB group: 24.6%) (Table 1, Supplementary Materrial 2). After PS matching, all baseline characteristics became well balanced between the treatment groups (Table 1, Supplementary Materrial 3). Among matched patients, 68.7% were aged 40-64 years, and 62.6% were male.

Over the 180-day follow-up period, most outcomes did not occur even once in either treatment group, making the calculation of HRs impossible for these outcomes. The risk of acute pancreatitis was not significantly different between patients receiving azilsartan medoxomil and amlodipine and those receiving other ARBs and amlodipine (summary HR, 0.86; 95% CI, 0.14 to 5.37). Specifically, in the HIRA database, during the 180-day follow-up period (mean duration: 98.0 days), there was 1 event of hypotension, 1 event of acute pancreatitis, 1 event of toxic liver disease, 6 events of nausea and vomiting, and no occurrences of other outcomes among patients receiving azilsartan medoxomil and amlodipine (Supplementary Material 4). After matching, the other ARB and amlodipine group experienced 2 events of hypotension, 1 angioedema event, 2 events of acute pancreatitis, 1 hyperkalemia event, 6 events of toxic liver disease, twelve events of nausea and vomiting, and 1 event of fall-related injury. The risks of hypotension, acute pancreatitis, toxic liver disease, and nausea and vomiting did not significantly differ between the azilsartan medoxomil and amlodipine and other ARB and amlodipine groups (HR, 1.00; 95% CI, 0.09 to 11.50; HR, 0.77; 95% CI, 0.07 to 8.88; HR, 0.50; 95% CI, 0.06 to 4.32; and HR, 1.01; 95% CI, 0.38 to 2.71, respectively). In the NHIRD database, risks of acute pancreatitis, hypokalemia, and fall-related injury were not significantly different between the 2 groups (HR, 1.00; 95% CI, 0.06 to 15.99; HR, 1.33; 95% CI, 0.30 to 5.96; and HR, 1.50; 95% CI, 0.53 to 4.21, respectively). HRs for other outcomes could not be calculated because no events occurred in the azilsartan treatment group (Table 2).

In both HIRA and NHIRD, risks of all outcomes did not differ when stratified by the initial dose of azilsartan medoxomil (Supplementary Material 5). These findings remained consistent when we applied PS fine stratification methods to adjust for confounders and when using the intention-to-treat analysis (Supplementary Materials 6 and 7). Due to limited sample sizes at both study sites, HRs for outcomes among patients who initiated concomitant high-dose azilsartan medoxomil (80 mg) and high-dose amlodipine (10 mg) could not be calculated (Supplementary Material 8). Our primary findings were also consistent in sensitivity analyses using olmesartan as an alternative active comparator rather than other ARBs (Supplementary Material 9).

## DISCUSSION

In this study, utilizing healthcare databases from Korea and Taiwan, we found no evidence suggesting an association between combined azilsartan medoxomil and amlodipine therapy and safety outcomes, including hypotension, angioedema, acute pancreatitis, hyperkalemia, hypokalemia, toxic liver disease, hepatic failure, nausea and vomiting, or fall-related injury. The incidence rates of all evaluated safety outcomes following initiation of azilsartan medoxomil and amlodipine were low at both study sites.

Several previous studies have examined the short-term safety profile of combined azilsartan medoxomil and amlodipine therapy. Weber et al. [18] conducted a 6-week RCT evaluating the safety of different doses of azilsartan medoxomil combined with amlodipine. The adverse event rates were comparable between the placebo with amlodipine (5 mg) group (47%) and the azilsartan medoxomil (40 mg) with amlodipine (5 mg) group (48%), and were even lower in the azilsartan medoxomil (80 mg) with amlodipine (5 mg) group (40%). Additionally, 4 participants experienced serious adverse events, 1 of which was syncope related to treatment with azilsartan medoxomil (40 mg) combined with amlodipine (5 mg), leading to study withdrawal. Another non-inferiority trial comparing azilsartan medoxomil and amlodipine combination therapy with telmisartan and amlodipine over 12 weeks found headaches were the most frequently reported side effect in both treatment groups, with no serious adverse events reported in either group [17].

Concerns about falls are common among patients with hypertension, particularly elderly individuals, and these concerns may influence decisions regarding antihypertensive treatment selection [26,27]. Previous studies examining the impact of various antihypertensive medications on fall risk indicated that ARBs generally do not increase this risk [27,28]. However, it remains uncertain whether the newer and more potent ARB, azilsartan medoxomil, could increase fall risk, especially given the possibility of increased risk associated with intensified antihypertensive therapy [27]. Our findings contribute to the existing literature by demonstrating that combination therapy with azilsartan medoxomil and amlodipine did not increase the risk of fall-related injuries compared to other ARBs combined with amlodipine.

The combination of azilsartan medoxomil and amlodipine has previously demonstrated potent antihypertensive effects [17,18]. One RCT showed blood pressure reductions of approximately 25/15 mmHg after 6 weeks with both 40 mg and 80 mg once-daily azilsartan medoxomil combined with amlodipine, significantly greater than the 14/8 mmHg reduction observed with placebo and amlodipine (p≤0.001) [18]. Another RCT demonstrated that after 12 weeks of treatment, the response rate with azilsartan medoxomil and amlodipine was non-inferior to that of telmisartan and amlodipine (88 vs. 96%, respectively; p=0.61) [17]. Although previous studies have documented the safety of combining other renin-angiotensin-aldosterone system inhibitors with amlodipine [29,30], direct head-to-head comparisons assessing the safety of combining the high-potency ARB azilsartan medoxomil with amlodipine remain lacking [14,15]. Thus, our study provides valuable real-world evidence supporting the safety of this combination, potentially informing future regulatory approval decisions.

This study has several notable strengths. First, by using 2 representative healthcare databases and employing a distributed network approach with a standardized protocol, the reliability and generalizability of our findings are enhanced. Second, our active comparator user study design minimizes susceptibility to confounding by indication and depletion of susceptibles bias [31,32].

Nevertheless, this study also has limitations. First, residual confounding may still exist, although we minimized potential bias by extensively adjusting for 43 baseline characteristics through PS matching. Second, limited sample sizes prevented the calculation of HRs for certain safety outcomes. While the low absolute numbers suggest that these outcomes were likely unrelated to azilsartan medoxomil and amlodipine therapy, further studies with larger populations are necessary to confirm these findings. Third, potential outcome misclassification might have influenced results. However, our focus on severe adverse events, which are typically distinct and more easily identified, makes such misclassification unlikely. Fourth, we assessed safety outcomes over a 6-month period, as ARB-related side effects typically manifest during the initial treatment months [14,33]. However, the 180-day follow-up may be insufficient for capturing longer-term safety issues; thus, these findings should be interpreted cautiously and require further investigation. Lastly, underestimation of some outcomes was possible. For example, nausea and vomiting often resolve spontaneously, and only severe cases usually result in medical encounters, meaning mild-to-moderate episodes might not have been captured. Similarly, hypotension frequently occurs asymptomatically, usually leading only to dose adjustments rather than medical encounters. Hence, captured events may represent particularly severe or incidental cases, and true incidence could be higher. Nonetheless, given comparable overall safety among ARBs, the risks likely remain non-differential between the compared groups.

In conclusion, this population-based cohort study found no evidence of an association between combined azilsartan medoxomil and amlodipine therapy and adverse safety outcomes—such as hypotension, angioedema, acute pancreatitis, hyperkalemia, hypokalemia, toxic liver disease, hepatic failure, nausea and vomiting, or fall-related injury—compared to therapy with other ARBs combined with amlodipine. Additionally, high-dose azilsartan medoxomil appeared safe in combination with regular or high-dose amlodipine. Our findings offer important real-world safety evidence for this therapeutic combination.

## Figures and Tables

**Figure f3-epih-47-e2025029:**
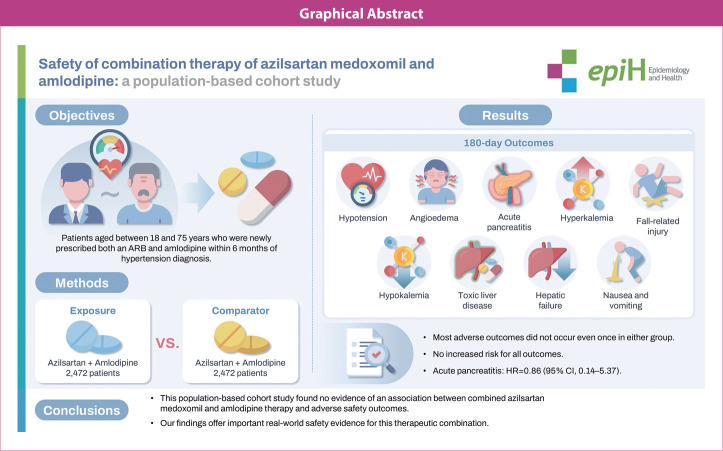


**Table 1.** Baseline characteristics before and after propensity score matching

Characteristics	Before matching			After matching		
	Azilsartan+ amlodipine (n=2,472)	Other ARB^1^+ amlodipine (n=671,468)	aSD	Azilsartan+ amlodipine (n=2,472)	Other ARB^1^+ amlodipine (n=2,472)	aSD
Age (yr)						
18-39	374 (15.1)	80,160 (11.9)	0.11	374 (15.1)	386 (15.6)	0.05
40-64	1,684 (68.1)	482,159 (71.8)	0.04	1,684 (68.1)	1,714 (69.4)	0.01
65-75	414 (16.7)	109,149 (16.3)	0.02	414 (16.7)	372 (15.0)	0.04
Sex						
Male	1,536 (62.2)	436,246 (65.0)	0.05	1,536 (62.2)	1,558 (63.0)	0.02
Female	936 (37.8)	235,222 (35.0)	0.05	936 (37.8)	914 (37.0)	0.02
Hospital level						
Tertiary general/general hospital	1,263 (51.1)	165,322 (24.6)	0.66	938 (38.0)	933 (37.7)	0.02
Others	1,209 (48.9)	506,146 (75.4)	0.66	1,534 (62.0)	1,539 (62.3)	0.02
Insurance type						
National Health Insurance	2,240 (99.0)	587,315 (99.0)	0.00	2,240 (99.0)	2,240 (99.0)	0.00
Medical Aid	43 (1.0)	9,341 (1.0)	0.00	43 (1.0)	41 (1.0)	0.00
CCI						
0	1,881 (76.1)	531,480 (79.1)	0.07	1,158 (74.9)	1,173 (74.8)	0.00
1	234 (9.5)	56,757 (8.5)	0.03	248 (10.0)	250 (10.1)	0.01
2	272 (11.0)	65,733 (9.8)	0.02	237 (9.6)	238 (9.6)	0.00
≥3	85 (3.4)	17,498 (2.6)	0.06	85 (3.4)	81 (3.3)	0.01
Comorbidities (general)						
Acute respiratory illness	715 (28.9)	214,313 (31.9)	0.07	715 (28.9)	716 (29.0)	0.00
Chronic liver disease	198 (8.0)	49,566 (7.4)	0.02	198 (8.0)	177 (7.2)	0.03
COPD	79 (3.2)	24,285 (3.6)	0.02	79 (3.2)	90 (3.6)	0.02
Diabetes	247 (10.0)	76,619 (11.4)	0.05	247 (10.0)	242 (9.8)	0.01
Gastroesophageal reflux disease	240 (9.7)	52,831 (7.9)	0.06	240 (9.7)	239 (9.7)	0.00
Gastrointestinal bleeding	53 (2.1)	17,039 (2.5)	0.03	53 (2.1)	50 (2.0)	0.01
Hyperlipidemia	497 (20.1)	140,191 (20.9)	0.02	497 (20.1)	485 (19.6)	0.01
Malignancy	97 (3.9)	24,081 (3.6)	0.02	97 (3.9)	111 (4.5)	0.03
Obesity	20 (0.8)	3,834 (0.6)	0.02	20 (0.8)	23 (0.9)	0.01
Osteoarthritis	267 (10.8)	57,858 (8.6)	0.07	267 (10.8)	269 (10.9)	0.00
Pneumonia	37 (1.5)	11,828 (1.8)	0.02	37 (1.5)	45 (1.8)	0.02
Psoriasis	17 (0.7)	3,018 (0.4)	0.04	17 (0.7)	17 (0.7)	0.00
Kidney disease	77 (3.1)	17,968 (2.7)	0.02	77 (3.1)	83 (3.4)	0.02
Rheumatoid arthritis	8 (0.3)	3,202 (0.5)	0.03	8 (0.3)	9 (0.4)	0.02
Ulcerative colitis	0 (0)	361 (0.1)	0.04	0 (0)	0 (0)	0.00
Urinary tract infections	35 (1.4)	15,606 (2.3)	0.07	35 (1.4)	46 (1.9)	0.04
Visual system disorder	519 (21.0)	120,866 (18.0)	0.08	519 (21.0)	499 (20.2)	0.02
Comorbidities (cardiovascular)						
Atrial fibrillation	11 (0.4)	2,216 (0.3)	0.02	11 (0.4)	11 (0.4)	0.00
Cerebrovascular disease	137 (5.5)	25,871 (3.9)	0.08	137 (5.5)	145 (5.9)	0.02
Coronary arteriosclerosis	28 (1.1)	11,502 (1.7)	0.05	28 (1.1)	25 (1.0)	0.01
Peripheral vascular disease	22 (0.9)	8,472 (1.3)	0.04	22 (0.9)	23 (0.9)	0.00
Pulmonary embolism	2 (0.1)	169 (0)	0.04	2 (0.1)	2 (0.1)	0.00
Venous thromboembolism	11 (0.4)	3,862 (0.6)	0.03	11 (0.4)	10 (0.4)	0.00
Use of medications						
Systemic antibacterials	840 (34.0)	221,585 (33.0)	0.02	840 (34.0)	838 (33.9)	0.00
Antidepressants	130 (5.3)	28,342 (4.2)	0.05	130 (5.3)	131 (5.3)	0.00
Antiepileptics	100 (4.0)	24,219 (3.6)	0.02	100 (4.0)	103 (4.2)	0.01
Anti-inflammatory and antirheumatic drugs	1,149 (46.5)	319,690 (47.6)	0.02	1,149 (46.5)	1,151 (46.6)	0.00
Antineoplastic drugs	NA^2^	2,210 (0.3)	NA^2^	NA^2^	NA^2^	NA^2^
Antithrombotic drugs	212 (8.6)	65,946 (9.8)	0.04	212 (8.6)	200 (8.1)	0.02
Beta blockers	398 (16.1)	89,306 (13.3)	0.08	398 (16.1)	403 (16.3)	0.01
Drugs for acid-related disorders	1,062 (43)	286,928 (42.7)	0.01	1,062 (43.0)	1,065 (43.1)	0.00
Drugs used for airway obstruction	344 (13.9)	109,019 (16.2)	0.06	344 (13.9)	334 (13.5)	0.01
Antidiabetic drugs	183 (7.4)	56,479 (8.4)	0.04	183 (7.4)	181 (7.3)	0.00
Immunosuppressants	NA^2^	3,249 (0.5)	NA^2^	NA^2^	9 (0.4)	NA^2^
Lipid-modifying agents	63 (2.5)	16,403 (2.4)	0.01	63 (2.5)	53 (2.1)	0.03
Opioids	355 (14.4)	70,057 (10.4)	0.12	355 (14.4)	343 (13.9)	0.01
Psycholeptics	504 (20.4)	140,418 (20.9)	0.01	504 (20.4)	501 (20.3)	0.00
Psychostimulants	29 (1.2)	6,780 (1.0)	0.02	29 (1.2)	32 (1.3)	0.01

Values are presented as number (%). aSD, absolute standardized difference; ARB, angiotensin receptor blockers; CCI, Charlson comorbidity index; COPD, chronic obstructive lung disease; NA, not applicable.

1 Other ARBs included all types of ARBs except for azilsartan.

2 Due to privacy issues in Taiwan, the exact number cannot be retrieved if the event number is less than 4 and thus not applicable.

**Table 2.** Propensity score-matched HRs of safety outcomes comparing the azilsartan + amlodipine group versus the other ARB^1^+amlodipine group after applying as-treated analysis^2^

Outcomes	Source	No. of events/Total, n (%)^3^		HR (95% CI)
		Azilsartan+amlodipine	Other ARB^1^+amlodipine	
Hypotension	HIRA	1/1,521 (0.07)	2/1,521 (0.13)	1.00 (0.09, 11.50)
	NHIRD	0/951 (0)	≤3/951 (NA)	NA
	Overall	1/2,472 (0.04)	NA	NA
Angioedema	HIRA	0/1,521 (0)	1/1,521 (0.07)	NA
	NHIRD	0/951 (0)	0/951 (0)	NA
	Overall	0/2,472 (0)	1/2,472 (0.04)	NA
Acute pancreatitis	HIRA	1/1,521 (0.07)	2/1,521 (0.13)	0.77 (0.07, 8.88)
	NHIRD	≤3/951 (NA)	≤3/951 (NA)	1.00 (0.06, 15.99)
	Overall	NA	NA	0.86 (0.14, 5.37)
Hyperkalemia	HIRA	0/1,521 (0)	1/1521 (0.07)	NA
	NHIRD	≤3/951 (NA)	≤3/951 (NA)	NA
	Overall	NA	NA	NA
Hypokalemia	HIRA	0/1,521 (0)	0/1,521 (0)	NA
	NHIRD	4/951 (0.42)	12/951 (1.26)	1.33 (0.30, 5.96)
	Overall	4/2,472 (0.16)	12/2,472 (0.49)	NA
Toxic liver disease	HIRA	1/1,521 (0.07)	6/1,521 (0.39)	0.50 (0.06, 4.32)
	NHIRD	0/951 (0)	0/951 (0)	NA
	Overall	1/2,472 (0.04)	6/2,472 (0.24)	NA
Hepatic failure	HIRA	0/1,521 (0)	0/1,521 (0)	NA
	NHIRD	≤3/951 (NA)	6/951 (0.63)	NA
	Overall	NA	6/2,472 (0.24)	NA
Nausea and vomiting	HIRA	6/1,521 (0.39)	12/1,521 (0.78)	1.01 (0.38, 2.71)
	NHIRD	0/951 (0)	≤3/951 (NA)	NA
	Overall	6/2,472 (0.24)	NA	NA
Fall-related injury	HIRA	0/1,521 (0)	1/1,521 (0.07)	NA
	NHIRD	9/951 (0.95)	19/951 (2.00)	1.50 (0.53, 4.21)
	Overall	9/2,472 (0.36)	20/2,472 (0.81)	NA

ARB, angiotensin receptor blockers; HR, hazard ratio; CI, confidence interval; HIRA, Health Insurance Review and Assessment Service; NHIRD, National Health Insurance Research Database; NA, not applicable.

1 Other ARBs included all types of ARBs except for azilsartan.

2 Patients were followed up from index date until the occurrence of study outcome, switching to another group, discontinuation (either ARB or amlodipine), death, or 180 days following index date, whichever occurs first.

3 Due to privacy issues in Taiwan, the exact number cannot be retrieved if the event number is less than 4 and thus not applicable.

## Supplementary materials

Supplementary Material 1. The definitions of outcomes, comorbidities and comedications.

Supplementary Material 2. Baseline characteristics before and after propensity score matching in HIRA.

Supplementary Material 3. Baseline characteristics before and after propensity score matching in NHIRD.

Supplementary Material 4. Crude hazard ratios of safety outcomes comparing azilsartan + amlodipine group versus other ARB^*^ + amlodipine group after applying as-treated analysis^†^.

Supplementary Material 5. Subgroup analyses stratified by azilsartan dose comparing safety outcomes of azilsartan + amlodipine group versus other ARB^*^ + amlodipine.

Supplementary Material 6. Propensity score fine stratification weighted hazard ratios of safety outcomes comparing azilsartan + amlodipine group versus other ARB^*^ + amlodipine group after applying as-treated analysis^†^.

Supplementary Material 7. Propensity score matched hazard ratios of safety outcomes comparing azilsartan + amlodipine group versus other ARB^*^ + amlodipine group after applying intention-to-treat analysis^†^.

Supplementary Material 8. Propensity score matched hazard ratios of safety outcomes comparing high-dose azilsartan plus high-dose amlodipine group versus other ARB^*^ plus amlodipine group after applying as-treated analysis^†^.

Supplementary Material 9. Propensity score matched hazard ratios of safety outcomes comparing azilsartan plus amlodipine group versus olmesartan plus amlodipine group after applying as-treated analysis^†^.
