# Supplementary material for: Safety of combination therapy of azilsartan medoxomil and amlodipine: a population-based cohort study
Source: Epidemiol Health. 2025 May 28;47:e2025029. doi: 10.4178/epih.e2025029 (PMC12425867; doi:10.4178/epih.e2025029)
Supplement: Supplementary Material 1. — The definitions of outcomes, comorbidities and comedications. [file epih-47-e2025029-Supplementary-1.docx]

**Supplementary Material 1.** The definitions of outcomes, comorbidities and comedications.

| **Variable** | **Role** | **Operational definition* (ICD-10)** | **Operational definition* (ICD-9)** |
| --- | --- | --- | --- |
| Safety outcomes | | | |
| Hypotension | Outcome | I95 | 458 |
| Angioedema | Outcome | T78.3 | 995.1 |
| Acute pancreatitis | Outcome | K85 | 577.0 |
| Hyperkalemia | Outcome | E87.5 | 276.7 |
| Hypokalemia | Outcome | E87.6 | 276.8 |
| Toxic liver disease | Outcome | K71 | 573.3 |
| Hepatic failure | Outcome | K72.0, K72.1, K72.9 | 570,572.8 |
| Nausea and vomiting | Outcome | R11 | 787.0 |
| Fall-related injury | Outcome | W00-W19 | E880-E888 |
| Hypotension | Outcome | I95 | 458 |
| Comorbidities (general) | | | |
| Acute respiratory disease | Comorbidity | J01-J05, J06.0, J06.9 | 460-466 |
| Chronic liver disease | Comorbidity | B18, I85.x, I86.4, I98.2, K70, K73-K77, Z94.4 | 571 |
| Chronic obstructive lung disease | Comorbidity | J43, J44, J45, J46 | 490-496 |
| Diabetes | Comorbidity | E10-E14 | 250 |
| Gastroesophageal reflux disease | Comorbidity | K21 | 530.81 |
| Gastrointestinal hemorrhage | Comorbidity | K22.6, K25.0, K25.2, K25.4, K25.6, K26.0, K26.2, K26.4, K26.6, K27.0, K27.2, K27.4, K27.6, K28.0, K28.2, K28.4, K28.6, K29.0, K62.5, K92.0, K92.1, K92.2 | 578 |
| Hyperlipidemia | Comorbidity | E78 | 272 |
| Malignancy | Comorbidity | C00-C43, C45-C97 | 140-239 |
| Obesity | Comorbidity | E660, E662, E663, E668, E669 | 278.0 |
| Osteoarthritis | Comorbidity | M15-M19 | 715 |
| Pneumonia | Comorbidity | J18 | 480-488 |
| Psoriasis | Comorbidity | L40 | 696.1 |
| Renal impairment | Comorbidity | E112, E132, E142, I12, I13, N00-N08, N17, N18, N19, N25-N27, Z49.0-Z49.2, Z94.0, Z99.2 | 580-589 |
| Rheumatoid arthritis | Comorbidity | M06 | 714.0 |
| Ulcerative colitis | Comorbidity | K51 | 556 |
| Urinary tract infections | Comorbidity | N39.0 | 599.0 |
| Visual system disorder | Comorbidity | H00-H59 | 360-379 |
| Medical history (cardiovascular disease) | | | |
| Atrial fibrillation | Comorbidity | I48 | 427.3 |
| Cerebrovascular disease | Comorbidity | I60-I69, G45(excl G45.4), G46 | 430-438 |
| Coronary artery sclerosis | Comorbidity | I25.1, I25.7 | 414.0 |
| Peripheral vascular disease | Comorbidity | I73.9 | 443.9 |
| Pulmonary embolism | Comorbidity | I26 | 415.1 |
| Venous thrombosis | Comorbidity | I80.0-I80.3, I80.8-I80.9, I82.9, O22.2 – O22.3, O87.0 –O87.1, I26.0, and I26.9. | 451-456 |
| Systemic antibacterials | Comedication | J01 | J01 |
| Antidepressants | Comedication | N06A | N06A |
| Antiepileptics | Comedication | N03 | N03 |
| Anti-inflammatory and antirheumatic drugs | Comedication | M01 | M01 |
| Antineoplastic drugs | Comedication | L01 | L01 |
| Antithrombotic drugs | Comedication | B01A | B01A |
| Beta blockers | Comedication | C07, C09BX02, C09BX04 | C07, C09BX02, C09BX04 |
| Drugs for acid-related disorders | Comedication | A02 | A02 |
| Drugs used for airway obstruction | Comedication | R03 | R03 |
| Antidiabetic drugs | Comedication | A10 | A10 |
| Immunosuppressants | Comedication | L04 | L04 |
| Lipid-lowering agents | Comedication | C10AB, C10AC, C10AD, C10AX | C10AB, C10AC, C10AD, C10AX |
| Opioids | Comedication | Alfentanil, buprenorphine, butorphanol, fentanyl, hydromorphone, morphine, nalbuphine, naloxone, oxycodone, pentazocine, pethidine, remifentanil, sufentanil, tapentadol, tramadol | Alfentanil, buprenorphine, butorphanol, fentanyl, hydromorphone, morphine, nalbuphine, naloxone, oxycodone, pentazocine, pethidine, remifentanil, sufentanil, tapentadol, tramadol |
| Psycholeptics | Comedication | N05 | N05 |
| Psychostimulants | Comedication | N06B | N06B |
| Abbreviations: ICD, International Classification of Diseases.  * Comorbidities were identified using ICD-10 or ICD-9 code and comedications were identified using ATC code except for opioids. Opioids were identified using specific ingredients. | | | |
