# Supplementary material for: Safety of combination therapy of azilsartan medoxomil and amlodipine: a population-based cohort study
Source: Epidemiol Health. 2025 May 28;47:e2025029. doi: 10.4178/epih.e2025029 (PMC12425867; doi:10.4178/epih.e2025029)
Supplement: Supplementary Material 2. — Baseline characteristics before and after propensity score matching in HIRA. [file epih-47-e2025029-Supplementary-2.docx]

**Supplementary Material 2.** Baseline characteristics before and after propensity score matching in HIRA.

|  | Before matching | | |  | After matching | | |
| --- | --- | --- | --- | --- | --- | --- | --- |
|  | Azilsartan + amlodipine (n=1,521) | Other ARB* + amlodipine (n=312,911) | aSD |  | Azilsartan + amlodipine (n=1,521) | Other ARB* + amlodipine (n=1,521) | aSD |
| Age group (years) |  |  | 0.13 |  |  |  | 0.05 |
| 18-39 | 218 (14.3) | 33813 (10.8) |  |  | 218 (14.3) | 212 (13.9) |  |
| 40-64 | 1030 (67.7) | 228374 (73.0) |  |  | 1030 (67.7) | 1061 (69.8) |  |
| 65-75 | 273 (18.0) | 50724 (16.2) |  |  | 273 (18.0) | 248 (16.3) |  |
| Sex |  |  | 0.06 |  |  |  | 0.00 |
| Male | 947 (62.3) | 210789 (67.4) |  |  | 947 (62.3) | 950 (62.5) |  |
| Female | 574 (37.7) | 102122 (32.6) |  |  | 574 (37.7) | 571 (37.5) |  |
| Hospital level |  |  | 0.59 |  |  |  | 0.00 |
| Tertiary general/general hospital | 625 (41.1) | 49222 (15.7) |  |  | 625 (41.1) | 624 (41.0) |  |
| Others | 896 (58.9) | 263689 (84.3) |  |  | 896 (58.9) | 897 (59.0) |  |
| Insurance type |  |  | 0.11 |  |  |  | 0.01 |
| National health insurance | 1478 (97.2) | 303570 (97.0) |  |  | 1478 (97.2) | 1480 (97.3) |  |
| Medical aid | 43 (2.8) | 9341 (3.0) |  |  | 43 (2.8) | 41 (2.7) |  |
| CCI, n (%) |  |  | 0.08 |  |  |  | 0.03 |
| 0 | 1100 (72.3) | 237215 (75.8) |  |  | 1100 (72.3) | 1099 (72.3) |  |
| 1 | 175 (11.5) | 31833 (10.2) |  |  | 175 (11.5) | 170 (11.2) |  |
| 2 | 199 (13.1) | 36606 (11.7) |  |  | 199 (13.1) | 210 (13.8) |  |
| ≥3 | 47 (3.1) | 7257 (2.3) |  |  | 47 (3.1) | 42 (2.8) |  |
| Comorbidities (general) |  |  |  |  |  |  |  |
| Acute respiratory illness | 349 (23.0) | 66525 (21.3) | 0.04 |  | 349 (23.0) | 344 (22.6) | 0.01 |
| Chronic liver disease | 137 (9.0) | 27310 (8.7) | 0.01 |  | 137 (9.0) | 128 (8.4) | 0.02 |
| COPD | 54 (3.6) | 8702 (2.8) | 0.04 |  | 54 (3.6) | 56 (3.7) | 0.01 |
| Diabetes | 124 (8.2) | 26156 (8.4) | 0.01 |  | 124 (8.2) | 116 (7.6) | 0.02 |
| Gastroesophageal reflux disease | 199 (13.1) | 39288 (12.6) | 0.02 |  | 199 (13.1) | 203 (13.4) | 0.01 |
| Gastrointestinal bleeding | 10 (0.7) | 1709 (0.6) | 0.01 |  | 10 (0.7) | 6 (0.4) | 0.04 |
| Hyperlipidemia | 325 (21.4) | 67298 (21.5) | 0.00 |  | 325 (21.4) | 317 (20.8) | 0.01 |
| Malignancy | 36 (2.4) | 7066 (2.3) | 0.01 |  | 36 (2.4) | 46 (3.0) | 0.04 |
| Obesity | 3 (0.2) | 313 (0.1) | 0.03 |  | 3 (0.2) | 2 (0.1) | 0.02 |
| Osteoarthritis | 197 (13.0) | 31791 (10.2) | 0.09 |  | 197 (13.0) | 202 (13.3) | 0.01 |
| Pneumonia | 14 (0.9) | 2094 (0.7) | 0.03 |  | 14 (0.9) | 15 (1.0) | 0.01 |
| Psoriasis | 9 (0.6) | 1433 (0.5) | 0.02 |  | 9 (0.6) | 10 (0.7) | 0.01 |
| Kidney disease | 31 (2.0) | 3381 (1.1) | 0.08 |  | 31 (2.0) | 31 (2.0) | 0.00 |
| Rheumatoid arthritis | 8 (0.5) | 1388 (0.4) | 0.01 |  | 8 (0.5) | 9 (0.6) | 0.01 |
| Ulcerative colitis | 0 (0.0) | 216 (0.1) | 0.04 |  | 0 (0.0) | 0 (0.0) | 0.00 |
| Urinary tract infections | 7 (0.5) | 1428 (0.5) | 0.00 |  | 7 (0.5) | 9 (0.6) | 0.02 |
| Visual system disorder | 336 (22.1) | 56403 (18.0) | 0.10 |  | 336 (22.1) | 319 (21.0) | 0.03 |
| Comorbidities (cardiovascular) |  |  |  |  |  |  |  |
| Atrial fibrillation | 4 (0.3) | 743 (0.2) | 0.01 |  | 4 (0.3) | 5 (0.3) | 0.01 |
| Cerebrovascular disease | 75 (4.9) | 6940 (2.2) | 0.15 |  | 75 (4.9) | 75 (4.9) | 0.00 |
| Coronary arteriosclerosis | 6 (0.4) | 521 (0.2) | 0.04 |  | 6 (0.4) | 8 (0.5) | 0.02 |
| Peripheral vascular disease | 16 (1.1) | 7037 (2.3) | 0.09 |  | 16 (1.1) | 17 (1.1) | 0.01 |
| Pulmonary embolism | 2 (0.1) | 56 (0.0) | 0.04 |  | 2 (0.1) | 2 (0.1) | 0.00 |
| Venous thromboembolism | 3 (0.2) | 433 (0.1) | 0.01 |  | 3 (0.2) | 2 (0.2) | 0.02 |
| Use of medications |  |  |  |  |  |  |  |
| Systemic antibacterials | 462 (30.4) | 91397 (29.2) | 0.03 |  | 462 (30.4) | 445 (29.3) | 0.02 |
| Antidepressants | 83 (5.5) | 11104 (3.6) | 0.09 |  | 83 (5.5) | 79 (5.2) | 0.01 |
| Antiepileptics | 59 (3.9) | 9047 (2.9) | 0.05 |  | 59 (3.9) | 65 (4.3) | 0.02 |
| Anti-inflammatory and antirheumatic drugs | 638 (42.0) | 121518 (38.8) | 0.06 |  | 638 (42.0) | 638 (42.0) | 0.00 |
| Antineoplastic drugs | 6 (0.4) | 1019 (0.3) | 0.01 |  | 6 (0.4) | 8 (0.5) | 0.02 |
| Antithrombotic drugs | 122 (8.0) | 22831 (7.3) | 0.03 |  | 122 (8.0) | 114 (7.5) | 0.02 |
| Beta blockers | 188 (12.4) | 18614 (6.0) | 0.22 |  | 188 (12.4) | 196 (12.9) | 0.02 |
| Drugs for acid-related disorders | 734 (48.3) | 139621 (44.6) | 0.07 |  | 734 (48.3) | 734 (48.3) | 0.00 |
| Drugs used for airway obstruction | 144 (9.5) | 26941 (8.6) | 0.03 |  | 144 (9.5) | 130 (8.6) | 0.03 |
| Antidiabetic drugs | 85 (5.6) | 15429 (4.9) | 0.03 |  | 85 (5.6) | 82 (4.7) | 0.04 |
| Immunosuppressants | 9 (0.6) | 1313 (0.4) | 0.02 |  | 9 (0.6) | 9 (0.6) | 0.00 |
| Lipid-modifying agents | 50 (3.3) | 8294 (2.7) | 0.04 |  | 50 (3.3) | 43 (2.8) | 0.03 |
| Opioids | 298 (19.6) | 57390 (18.3) | 0.03 |  | 298 (19.6) | 295 (19.4) | 0.00 |
| Psycholeptics | 242 (15.9) | 41188 (13.2) | 0.08 |  | 242 (15.9) | 225 (14.8) | 0.03 |
| Psychostimulants | 15 (1.0) | 1312 (0.4) | 0.07 |  | 15 (1.0) | 19 (1.3) | 0.03 |
| Abbreviations: aSD, absolute standardized difference; ARB, angiotensin receptor blockers; CCI, charlson comorbidity score; COPD, chronic obstructive lung disease; HIRA, Health Insurance Review and Assessment Service.  *Other ARBs included all types of ARB except for azilsartan.  ^+^Due to privacy issues in Taiwan, the exact number cannot be retrieved if the event number is less than 4. | | | | | | | |
