# Supplementary material for: Safety of combination therapy of azilsartan medoxomil and amlodipine: a population-based cohort study
Source: Epidemiol Health. 2025 May 28;47:e2025029. doi: 10.4178/epih.e2025029 (PMC12425867; doi:10.4178/epih.e2025029)
Supplement: Supplementary Material 3. — Baseline characteristics before and after propensity score matching in NHIRD. [file epih-47-e2025029-Supplementary-3.docx]

**Supplementary Material 3.** Baseline characteristics before and after propensity score matching in NHIRD.

|  | Before matching | | |  | After matching | | |
| --- | --- | --- | --- | --- | --- | --- | --- |
|  | Azilsartan + amlodipine (n=951) | Other ARB* + amlodipine (n=358,557) | aSD |  | Azilsartan + amlodipine (n=951) | Other ARB* + amlodipine (n=951) | aSD |
| Age group (years) |  |  |  |  |  |  |  |
| 18-39 | 156 (16.4) | 46347 (12.9) | 0.1 |  | 156 (16.4) | 174 (18.3) | 0.05 |
| 40-64 | 654 (68.8) | 253785 (70.8) | 0.04 |  | 654 (68.8) | 653 (68.7) | 0.00 |
| 65-75 | 141 (14.8) | 58425 (16.3) | 0.04 |  | 141 (14.8) | 124 (13) | 0.05 |
| Sex |  |  | 0.02 |  |  |  | 0.04 |
| Male | 589 (61.9) | 225457 (62.9) |  |  | 589 (61.9) | 608 (63.9) |  |
| Female | 362 (38.1) | 133100 (37.1) |  |  | 362 (38.1) | 343 (36.1) |  |
| Hospital level |  |  | 0.74 |  |  |  | 0.02 |
| Tertiary general/general hospital | 638 (67.1) | 116100 (32.4) |  |  | 313 (32.9) | 309 (32.5) |  |
| Others | 313 (32.9) | 242457 (67.6) |  |  | 781 (82.1) | 769 (80.9) | 0.03 |
| Insurance type |  |  | 0.00 |  |  |  | 0.00 |
| National health insurance | 762(100.0) | 283745 (100.0) |  |  | 762(100.0) | 283745 (100.0) |  |
| Medical aid | 0 (0.0) | 0 (0.0) |  |  | 0 (0.0) | 0 (0.0) |  |
| CCI, n (%) |  |  |  |  |  |  |  |
| 0 | 781 (82.1) | 294265 (82.1) | 0.00 |  | 59 (6.2) | 74 (7.8) | 0.06 |
| 1 | 59 (6.2) | 24924 (7.0) | 0.03 |  | 73 (7.7) | 80 (8.4) | 0.03 |
| 2 | 73 (7.7) | 29127 (8.1) | 0.02 |  | 38 (4.0) | 28 (2.9) | 0.06 |
| ≥3 | 38 (4.0) | 10241 (2.9) | 0.06 |  | 38 (4.0) | 36 (3.8) | 0.01 |
| Comorbidities (general) |  |  |  |  |  |  |  |
| Acute respiratory illness | 366 (38.5) | 147788 (41.2) | 0.06 |  | 366 (38.5) | 372 (39.1) | 0.01 |
| Chronic liver disease | 61 (6.4) | 22256 (6.2) | 0.01 |  | 61 (6.4) | 49 (5.2) | 0.05 |
| COPD | 25 (2.6) | 15583 (4.3) | 0.09 |  | 25 (2.6) | 34 (3.6) | 0.06 |
| Diabetes | 123 (12.9) | 50463 (14.1) | 0.03 |  | 123 (12.9) | 126 (13.2) | 0.01 |
| Gastroesophageal reflux disease | 41 (4.3) | 13543 (3.8) | 0.03 |  | 41 (4.3) | 36 (3.8) | 0.03 |
| Gastrointestinal bleeding | 43 (4.5) | 15330 (4.3) | 0.01 |  | 43 (4.5) | 44 (4.6) | 0.01 |
| Hyperlipidemia | 172 (18.1) | 72893 (20.3) | 0.06 |  | 172 (18.1) | 168 (17.7) | 0.01 |
| Malignancy | 61 (6.4) | 17015 (4.7) | 0.07 |  | 61 (6.4) | 65 (6.8) | 0.02 |
| Obesity | 17 (1.8) | 3521 (1.0) | 0.07 |  | 17 (1.8) | 21 (2.2) | 0.03 |
| Osteoarthritis | 70 (7.4) | 26067 (7.3) | 0.00 |  | 70 (7.4) | 67 (7) | 0.01 |
| Pneumonia | 23 (2.4) | 9734 (2.7) | 0.02 |  | 23 (2.4) | 30 (3.2) | 0.05 |
| Psoriasis | 8 (0.8) | 1585 (0.4) | 0.05 |  | 8 (0.8) | 7 (0.7) | 0.01 |
| Kidney disease | 46 (4.8) | 14587 (4.1) | 0.04 |  | 46 (4.8) | 52 (5.5) | 0.03 |
| Rheumatoid arthritis | 0 (0.0) | 1814 (0.5) | 0.10 |  | 0 (0.0) | 0 (0.0) | 0.00 |
| Ulcerative colitis | 0 (0.0) | 145 (0) | 0.03 |  | 0 (0.0) | 0 (0.0) | 0.00 |
| Urinary tract infections | 28 (2.9) | 14178 (4) | 0.06 |  | 28 (2.9) | 37 (3.9) | 0.05 |
| Visual system disorder | 183 (19.2) | 64463 (18.0) | 0.03 |  | 183 (19.2) | 180 (18.9) | 0.01 |
| Comorbidities (cardiovascular) |  |  |  |  |  |  |  |
| Atrial fibrillation | 7 (0.7) | 1473 (0.4) | 0.04 |  | 7 (0.7) | 6 (0.6) | 0.01 |
| Cerebrovascular disease | 62 (6.5) | 18931 (5.3) | 0.05 |  | 62 (6.5) | 70 (7.4) | 0.03 |
| Coronary arteriosclerosis | 22 (2.3) | 10981 (3.1) | 0.05 |  | 22 (2.3) | 17 (1.8) | 0.04 |
| Peripheral vascular disease | 6 (0.6) | 1435 (0.4) | 0.03 |  | 6 (0.6) | 6 (0.6) | 0.00 |
| Pulmonary embolism | 0 (0.0) | 113 (0) | 0.03 |  |  |  |  |
| Venous thromboembolism | 8 (0.8) | 3429 (1.0) | 0.01 |  | 8 (0.8) | 8 (0.8) | 0.00 |
| Use of medications |  |  |  |  |  |  |  |
| Systemic antibacterials | 378 (39.7) | 130188 (36.3) | 0.07 |  | 378 (39.7) | 393 (41.3) | 0.03 |
| Antidepressants | 47 (4.9) | 17238 (4.8) | 0.01 |  | 47 (4.9) | 52 (5.5) | 0.02 |
| Antiepileptics | 41 (4.3) | 15172 (4.2) | 0 |  | 41 (4.3) | 38 (4) | 0.02 |
| Anti-inflammatory and antirheumatic drugs | 511 (53.7) | 198172 (55.3) | 0.03 |  | 511 (53.7) | 513 (53.9) | 0.00 |
| Antineoplastic drugs | ≤5 | 1191 (0.3) | 0.01 |  | ≤5 | ≤5 | 0.06 |
| Antithrombotic drugs | 90 (9.5) | 43115 (12.0) | 0.08 |  | 90 (9.5) | 86 (9.0) | 0.02 |
| Beta blockers | 210 (22.1) | 70692 (19.7) | 0.06 |  | 210 (22.1) | 207 (21.8) | 0.01 |
| Drugs for acid-related disorders | 328 (34.5) | 147307 (41.1) | 0.14 |  | 328 (34.5) | 331 (34.8) | 0.01 |
| Drugs used for airway obstruction | 200 (21) | 82078 (22.9) | 0.05 |  | 200 (21.0) | 204 (21.5) | 0.01 |
| Antidiabetic drugs | 98 (10.3) | 41050 (11.4) | 0.04 |  | 98 (10.3) | 99 (10.4) | 0.00 |
| Immunosuppressants | ≤5 | 1936 (0.5) | 0.00 |  | ≤5 | 0 (0) | 0.10 |
| Lipid-modifying agents | 13 (1.4) | 8109 (2.3) | 0.07 |  | 13 (1.4) | 10 (1.1) | 0.03 |
| Opioids | 57 (60) | 12667 (3.5) | 0.12 |  | 57 (6) | 48 (5.0) | 0.04 |
| Psycholeptics | 262 (27.5) | 99230 (27.7) | 0.00 |  | 262 (27.5) | 276 (29.0) | 0.03 |
| Psychostimulants | 14 (1.5) | 5468 (1.5) | 0.00 |  | 14 (1.5) | 13 (1.4) | 0.01 |
| Abbreviations: aSD, absolute standardized difference; ARB, angiotensin receptor blockers; CCI, charlson comorbidity score; COPD, chronic obstructive lung disease; NHIRD, National Health Insurance Research Database.  *Other ARBs included all types of ARB except for azilsartan.  ^+^Due to privacy issues in Taiwan, the exact number cannot be retrieved if the event number is less than 4. | | | | | | | |
