# Supplementary material for: Safety of combination therapy of azilsartan medoxomil and amlodipine: a population-based cohort study
Source: Epidemiol Health. 2025 May 28;47:e2025029. doi: 10.4178/epih.e2025029 (PMC12425867; doi:10.4178/epih.e2025029)
Supplement: Supplementary Material 5. — Subgroup analyses stratified by azilsartan dose comparing safety outcomes of azilsartan + amlodipine group versus other ARB* + amlodipine. [file epih-47-e2025029-Supplementary-5.docx]

**Supplementary Material 5.** Subgroup analyses stratified by azilsartan dose comparing safety outcomes of azilsartan + amlodipine group versus other ARB* + amlodipine.

| Outcomes | Dose of Azilsartan | Azilsartan + amlodipine versus Other ARB* + amlodipine |
| --- | --- | --- |
|  |  | Pooling hazard Ratio (95% CI) |
| Hypotension | Standard | NA |
|  | High | NA |
| Angioedema | Standard | NA |
|  | High | NA |
| Acute pancreatitis | Standard | NA |
|  | High | NA |
| Hyperkalemia | Standard | NA |
|  | High | NA |
| Hypokalemia | Standard | NA |
|  | High | NA |
| Toxic liver disease | Standard | NA |
|  | High | NA |
| Hepatic failure | Standard | NA |
|  | High | NA |
| Nausea and vomiting | Standard | 0.65 (0.23-1.84) |
|  | High | NA |
| Fall-related injury | Standard | NA |
|  | High | NA |
| Abbreviations: ARB, angiotensin receptor blockers; CI, confidence interval; NA, not applicable.  *Other ARBs included all types of ARB except for azilsartan. | | |
