# Supplementary material for: Safety of combination therapy of azilsartan medoxomil and amlodipine: a population-based cohort study
Source: Epidemiol Health. 2025 May 28;47:e2025029. doi: 10.4178/epih.e2025029 (PMC12425867; doi:10.4178/epih.e2025029)
Supplement: Supplementary Material 6. — Propensity score fine stratification weighted hazard ratios of safety outcomes comparing azilsartan + amlodipine group versus other ARB* + amlodipine group after applying as-treated analysis†. [file epih-47-e2025029-Supplementary-6.docx]

**Supplementary Material 6.** Propensity score fine stratification weighted hazard ratios of safety outcomes comparing azilsartan + amlodipine group versus other ARB* + amlodipine group after applying as-treated analysis†.

| Outcome | Source | No. of events/total No. | | Hazard Ratio (95% CI) |
| --- | --- | --- | --- | --- |
|  |  | Azilsartan + amlodipine | Other ARB^*^ + amlodipine |  |
| Hypotension | HIRA | 2/1521 | 218/312322 | 2.63 (0.74-37.63) |
|  | NHIRD | 0/951 | 631/355409 | NA |
|  | Overall | 2/2472 | 849/667731 | NA |
| Angioedema | HIRA | 0/1521 | 55/312322 | NA |
|  | NHIRD | 0/951 | 25/355409 | NA |
|  | Overall | 0/2472 | 80/667731 | NA |
| Acute pancreatitis | HIRA | 1/1521 | 206/312322 | 2.68 (0.37-19.19) |
|  | NHIRD | ≤10/951 | 1640/355409 | 1.79 (0.44-7.35) |
|  | Overall | NA | 1846/667731 | 2.12 (0.60-7.53) |
| Hyperkalemia | HIRA | 0/1521 | 76/312322 | NA |
|  | NHIRD | ≤10/951 | 1781/355409 | 1.23 (0.25-6.13) |
|  | Overall | NA | 1857/667731 | NA |
| Hypokalemia | HIRA | 0/1521 | 48/312322 | NA |
|  | NHIRD | ≤10/951 | 5107/355409 | 1.22 (0.46-3.20) |
|  | Overall | NA | 5155/667731 | NA |
| Toxic liver disease | HIRA | 2/1521 | 515/312322 | 1.65 (0.23-11.72) |
|  | NHIRD | 0/951 | 0/355409 | NA |
|  | Overall | 2/2472 | 515/667731 | NA |
| Hepatic failure | HIRA | 0/1521 | 34/312322 | NA |
|  | NHIRD | ≤10/951 | 1680/355409 | 0.81 (0.10-6.43) |
|  | Overall | NA | 1714/667731 | NA |
| Nausea and vomiting | HIRA | 5/1521 | 2899/312322 | 0.71 (0.27-1.86) |
|  | NHIRD | 0/951 | 419/355409 | NA |
|  | Overall | 5/2472 | 3318/667731 | NA |
| Fall-related injury | HIRA | 0/1521 | 43/312322 | NA |
|  | NHIRD | 15/951 | 12363/355409 | 2.03 (1.23-3.34) |
|  | Overall | 15/2472 | 12406/667731 | NA |
| Abbreviations: ARB, angiotensin receptor blockers; CI, confidence interval; HIRA, Health Insurance Review and Assessment Service; NA, not applicable; NHIRD, National Health Insurance Research Database.  ^*^Other ARBs included all types of ARB except for azilsartan.  †Patients were followed up from index date until the occurrence of study outcome, switching to another group, discontinuation (either ARB or amlodipine), death, or 180 days following index date, whichever occurs first.  Due to privacy issues in Taiwan, the exact number cannot be retrieved if the event number is less than 4 and thus not applicable. | | | | |
